# Circulating cell-free DNA has a high degree of specificity to detect exon 19 deletions and the single-point substitution mutation L858R in non-small cell lung cancer

**DOI:** 10.18632/oncotarget.8684

**Published:** 2016-04-11

**Authors:** Xin Qian, Jia Liu, Yuhui Sun, Meifang Wang, Huaiding Lei, Guoshi Luo, Xianjun Liu, Chang Xiong, Dan Liu, Jie Liu, Yijun Tang

**Affiliations:** ^1^ Department of Respiratory Medicine, Taihe Hospital, Hubei University of Medicine, Shiyan, 442000, Hubei, P.R. China; ^2^ Institute of Respiratory Medicine, Taihe Hospital, Hubei University of Medicine, Shiyan, 442000, Hubei, P.R. China; ^3^ Department of Orthopedic, Lanzhou University First Hospital, Lanzhou, 730000, Gansu, P.R. China; ^4^ Department of Emergency Medicine, Taihe Hospital, Hubei University of Medicine, Shiyan, 442000, Hubei, P.R. China

**Keywords:** circulating cell-free DNA, non-small cell lung cancer, sensitivity, specificity, epidermal growth factor receptor

## Abstract

Detection of an epidermal growth factor receptor (EGFR) mutation in circulating cell-free DNA (cfDNA) is a noninvasive method to collect genetic information to guide treatment of lung cancer with tyrosine-kinase inhibitors (TKIs). However, the association between cfDNA and detection of EGFR mutations in tumor tissue remains unclear. Here, a meta-analysis was performed to determine whether cfDNA could serve as a substitute for tissue specimens for the detection of EGFR mutations. The pooled sensitivity, specificity, and areas under the curve of cfDNA were 0.60, 0.94, and 0.9208 for the detection of EGFR mutations, 0.64, 0.99, and 0.9583 for detection of the exon 19 deletion, and 0.57, 0.99, and 0.9605 for the detection of the L858R mutation, respectively. Our results showed that cfDNA has a high degree of specificity to detect exon 19 deletions and L858R mutation. Due to its high specificity and noninvasive characteristics, cfDNA analysis presents a promising method to screen for mutations in NSCLC and predict patient response to EGFR-TKI treatment, dynamically assess treatment outcome, and facilitate early detection of resistance mutations.

## INTRODUCTION

Lung cancer is the leading cause of cancer-related death, accounting for more than 27% of all cancer deaths worldwide [1]. Lung cancer is classified as non-small cell lung cancer (NSCLC) (87% of cases) or small cell lung cancer (13% of cases) for the purpose of treatment [2]. Even with the recent advances in the treatment of lung cancers, the 5-year relative survival rate is currently 18%, as more than 50% of cases are diagnosed at an advanced stage [1].

Epidermal growth factor receptor (EGFR) is a receptor tyrosine kinase (TK). EGFR mutations lead to constitutive activation of downstream signaling pathways that promote cell proliferation [3]. EGFR mutations are present in 10% of NSCLC cases in North America and Europe, and more common (> 50%) among non-smokers, adenocarcinomas, and Asian patients [4]. The most commonly found mutations are in-frame deletions of amino acids 747–750 in exon 19 (exon 19 deletion), accounting for 45% of mutations, and exon 21 mutations resulting in the single-point substitution mutation L858R, which accounts for 40%–45% of such mutations [4]. Both exon 19 deletions and the L858R point mutation result in activation of the TK domain, and both are correlated with sensitivity to small molecule TK inhibitors (TKIs), such as erlotinib, gefitinib, and afatinib. Treatment with TKIs is correlated with a statistically significant and clinically meaningful response rate and prognosis [5]. Among wild-type EGFR patients, survival was superior in those who received first-line chemotherapy than those who received erlotinib first followed by subsequent chemotherapy (11.6 vs. 8.7 months, respectively), while the point mutation T790M in exon 20 is associated with poorer response and shorter survival [6, 7]. Thus, detection of EGFR mutation status is critical to determine an appropriate treatment strategy, especially for the administration of EGFR-TKIs as a first-line therapy. Additional studies have shown that different mutations are associated with varying clinical outcomes. For example, NSCLC harboring the EGFR exon 19 deletion may be more susceptible to TKIs as compared with tumors with the L858R mutation [8–10]. So, detection of EGFR mutation type is important to predict the effect of TKI treatment.

Currently, tumor tissue, which is usually obtained by biopsy or surgery, is the gold standard for detection of EGFR mutations. Unfortunately, most NSCLC patients are diagnosed at an advanced stage; thus, it is difficult to obtain tumor samples from non-operated patients. Additionally, sample preservation and tumor heterogeneity also hamper the use of tumor tissue for cancer sequencing, with different areas of the same tumor showing different genetic profiles (intratumor heterogeneity) [11]. So, genomic analysis from single tumor biopsy may underestimate the mutational burden of heterogeneous tumors [12]. Thus, development of new methods is needed for the detection of EGFR mutations in patients with little or no available tumor sample.

Circulating cell-free DNA (cfDNA) can provide the same genetic information as a tissue biopsy and can be drawn at any time during the course of therapy allowing for dynamic monitoring of molecular change [11]. Detection of EGFR mutations in blood may provide a noninvasive and replicable source of genetic information [11, 13]. Although, numerous studies have investigated the diagnostic accuracy of cfDNA for detection of EGFR mutations [14–17], the concordance rate of EGFR mutations between cfDNA and tumor tissue varies.

Therefore, we conducted this meta-analysis to investigate the diagnostic accuracy of cfDNA for detection of the two main EGFR mutations in tumor tissues in lung cancer.

## RESULTS

### Characteristics of eligible studies

**Figure 1:**
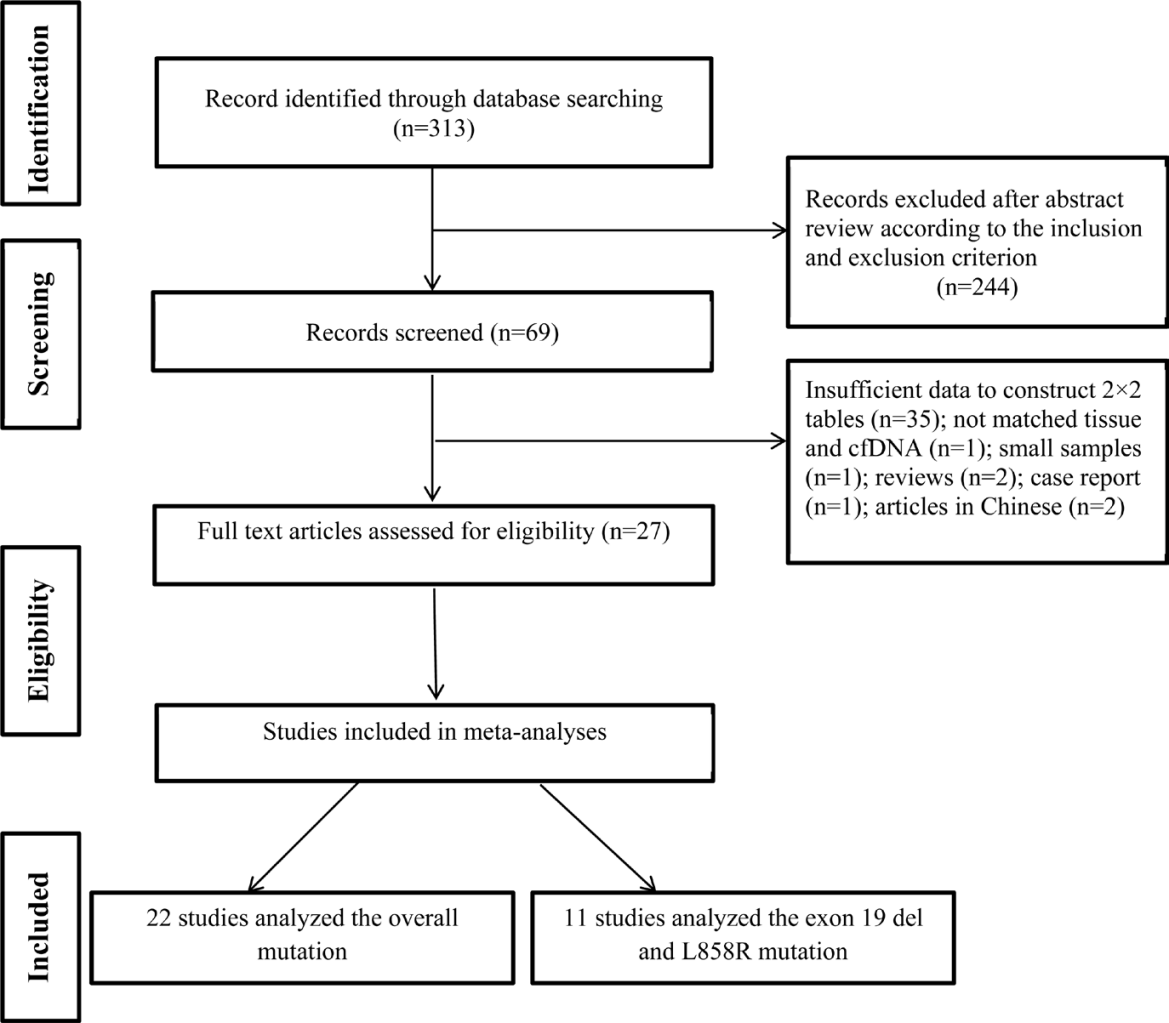
Flowchart of study selection

Of a total of 313 articles identified during the initial search, 244 were excluded after reviewing the titles and abstracts, leaving 69 articles for further analysis of the full text. Of these, 27 articles met the inclusion criteria, which included 22 studies of all EGFR mutations [14–16, 18–35]. Eleven articles were selected for the meta-analysis of the exon 19 deletion and the L858R point mutation [14–17, 20, 28, 36–39] (Figure 1). Of these, Li et al. [20] detected EGFR mutations both in plasma and serum, so the data from plasma and serum were analyzed independently. All included studies were published between 2007 and 2015. Three studies were conducted in Japan [14, 28, 33], two in Korea [23, 27], two in France [16, 36], one in Australia [31], one in the US [35], one in Spain [39], one in Denmark [19], and the others in China. Characteristics of the eligible studies are shown in Table 1.

**Table 1:** Characteristics of eligible studies

Author	Year	Country	Number	Female	Age	Ever smoker	AC	Method	Sample	TNM (I/II/III/IV/other)	All EGFR mutations				Exon 19 deletion				L858R point mutation			
											TP	FP	FN	TN	TP	FP	FN	TN	TP	FP	FN	TN
Lam D	2015	China	74	36	65 ± 12	25	72	PNA-LNA PCR	plasma	0/0/4/70	34	1	9	30								
Uchida J	2015	Japan	288	119	< 60 (66)	NR	274	Deep sequencing	plasma	64/19/53/146/6	56	22	47	163	27	5	26	230	26	14	26	222
Karachaliou N	2015	Spain	97	68	< 65 (45)	26	93	PNA clamp	serum	0/0/4/93					47	0	9	41	29	0	12	56
Mok T	2015	China	238	NR	NR	NR	NR	AS-PCR	plasma	NR	72	5	24	137	47	3	10	178	23	2	14	199
Zhu G	2015	China	86	30	55 (28-81)	47	85	ddPCR	plasma	0/0/4/82					18	1	4	63	12	3	3	68
Douillard J	2014	France	652	NR	NR	NR	NR	ARMS	plasma	NR	69	1	36	546	48	0	23	581	21	1	13	617
Couraud S	2014	France	59	NR	NR	NR	NR	NGS	plasma	NR					11	2	9	37	6	0	2	53
Weber B	2014	Denmark	196	NR	NR	NR	NR	AS-PCR	plasma	NR	17	6	11	162								
Li X (plasma)	2014	China	121	NR	NR	NR	NR	ARMS	plasma	NR	27	3	29	62	17	2	16	86	11	2	12	97
Li X (serum)	2014	China	92	NR	NR	NR	NR	ARMS	serum	NR	19	2	29	42	12	1	15	64	7	1	14	70
Wang S	2014	China	134	65	< 60 (90)	62	108	ARMS	plasma	0/0/19/115	15	2	53	64								
Jing C	2013	China	120	51	62 (36-85)	NR	70	HRM	plasma	38 (I/II)/ 82 (III/IV)	29	2	16	73								
Kim HR	2013	Korea	40	NR	NR	NR	NR	PNA	plasma	NR	6	0	29	5								
Kim ST	2013	Korea	57	22	64 (28-84)	32	40	PNA-LNA PCR	serum	NR	8	3	4	42								
Zhang H	2013	China	86	37	58 (21-80)	44	65	MEL	plasma	0/0/16/70					15	0	7	64	8	0	6	72
Liu X	2013	China	86	30	55 (28-81)	47	85	ARMS	plasma	0/0/4/82	27	0	13	46								
Hu C	2012	China	47	NR	NR	NR	28	HRM	serum	NR	22	0	2	23								
Zhao X	2012	China	111	35	< 60 (52)	57	73	ME-PCR	plasma	22/10/33/46	16	3	29	63								
Goto K	2012	Japan	86	NR	NR	NR	NR	ARMS	serum	NR	22	0	29	35	11	0	18	57	10	0	12	64
Xu F	2012	China	34	NR	NR	NR	NR	ARMS	serum	NR					3	4	4	23	4	0	4	26
Huang Z	2012	China	822	384	≤ 65 (519)	340	641	DHPLC	plasma	NR	188	81	108	445								
Jiang B	2011	China	58	18	56 (43-80)	36	42	ME-sequencing	serum	NR	14	0	4	40								
Sriram K	2011	Australia	64	NR	NR	NR	NR	ME-PCR	serum	NR	3	0	3	58								
He C	2009	China	18	NR	NR	NR	NR	ME-PCR	plasma	NR	8	1	0	9								
Bai H	2009	China	230	107	60.7 ± 4.5	103	171	DHPLC	plasma	0/0/80/150	63	16	14	137								
Kuang Y	2009	USA	NR	NR	NR	NR	NR	ARMS	plasma	NR	21	2	9	11								
Kimura H	2007	Japan	42	14	58 (40-81)	28	31	ARMS	serum	NR	6	1	2	33								

PCR: polymerase chain reaction; PNA-LNA PCR: peptide nucleic acid-locked nucleic acid PCR; AS-PCR: allele-specific PCR; ARMS: scorpion amplification refractory mutation system; HRM: high resolution melting; ddPCR: droplet digital PCR; PNA: peptide nucleic acid-mediated PCR clamping; MEL: mutant-enriched liquidchip; ME-PCR: mutant-enriched PCR; DHPLC: denaturing high-performance liquid chromatography; ME sequencing: mutant-enriched sequencing; NGS: next-generation sequencing; NR: not reported; AC: adenocarcinoma; TNM: tumor node metastasis;

TP: true positive; FP: false positive; FN: false negative; TN: true negative.

### Quality assessment of studies

QUADAS-2 was used to estimate the quality of each eligible study. As shown in Table 2, the methodological quality of the eligible studies was not significantly high.

**Table 2:** Quality assessment of 27 studies by QUADAS-2

Study	Risk of bias				Applicability concerns		
	Patient selection	Index test	Reference standard	Flow and timing	Patient selection	Index test	Reference standard
Lam D	L	UC	L	L	L	L	L
Uchida J	L	L	L	L	L	L	L
Karachaliou N	L	L	L	L	L	L	L
Mok T	L	UC	L	L	L	L	L
Zhu G	L	H	L	L	L	L	L
Douillard J	L	L	L	L	L	L	L
Couraud S	L	L	L	L	L	L	L
Weber B	L	UC	L	L	L	L	L
Li X (plasma)	L	L	L	L	L	L	L
Li X (serum)	L	L	L	L	L	L	L
Wang S	L	UC	L	L	L	L	L
Jing C	L	L	L	L	L	L	L
Kim HR	L	L	L	L	L	L	L
Kim ST	L	L	L	L	L	L	L
Zhang H	L	L	UC	L	L	L	L
Liu X	L	L	L	L	L	L	L
Hu C	L	L	UC	L	L	L	L
Zhao X	L	L	UC	L	L	L	L
Goto K	L	L	UC	L	L	L	L
Xu F	L	L	L	L	L	L	L
Huang Z	L	UC	L	L	L	L	L
Jiang B	L	L	L	L	L	L	L
Sriram K	L	H	L	L	L	L	L
He C	L	H	L	L	L	L	L
Bai H	L	L	L	L	L	L	L
Kuang Y	L	UC	L	L	L	L	L
Kimura H	L	H	L	L	L	L	L

L: Low H: High UC: Unclear.

### Publication bias and sensitivity analysis

**Figure 2:**
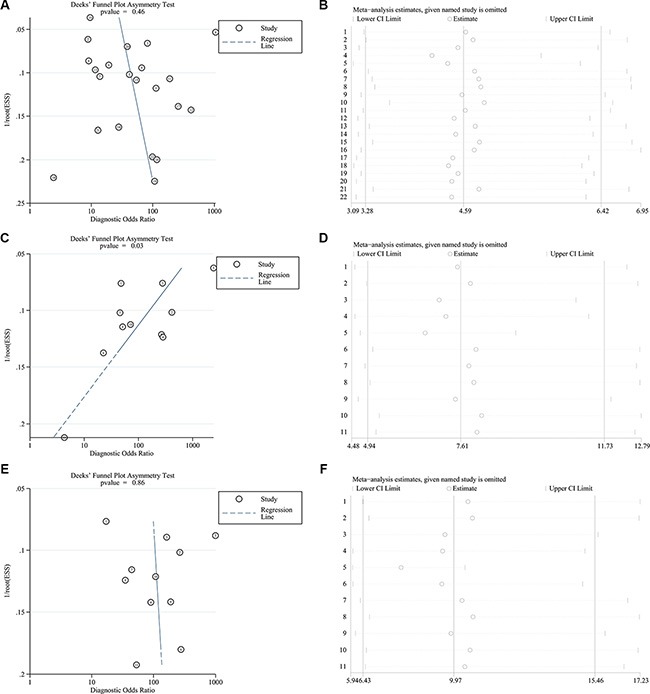
Deek's funnel plots and sensitivity analyses of all EGFR mutations (A, B), the exon 19 deletion (C, D), and the L858R point mutation (E, F) in the pooled studies

Deek's funnel plots and *p* values were used to estimate publication bias. As shown in Figure 2, the *p* values for all mutations and the L858R point mutation were 0.46 and 0.86, suggesting no significant publication bias, while the *p* value of the exon 19 deletion was 0.03, indicating the likelihood of publication bias. Thus, we conducted sensitivity analysis and found that the pooled results were not affected by individual studies (Figure 2).

### Overall analysis

**Figure 3:**
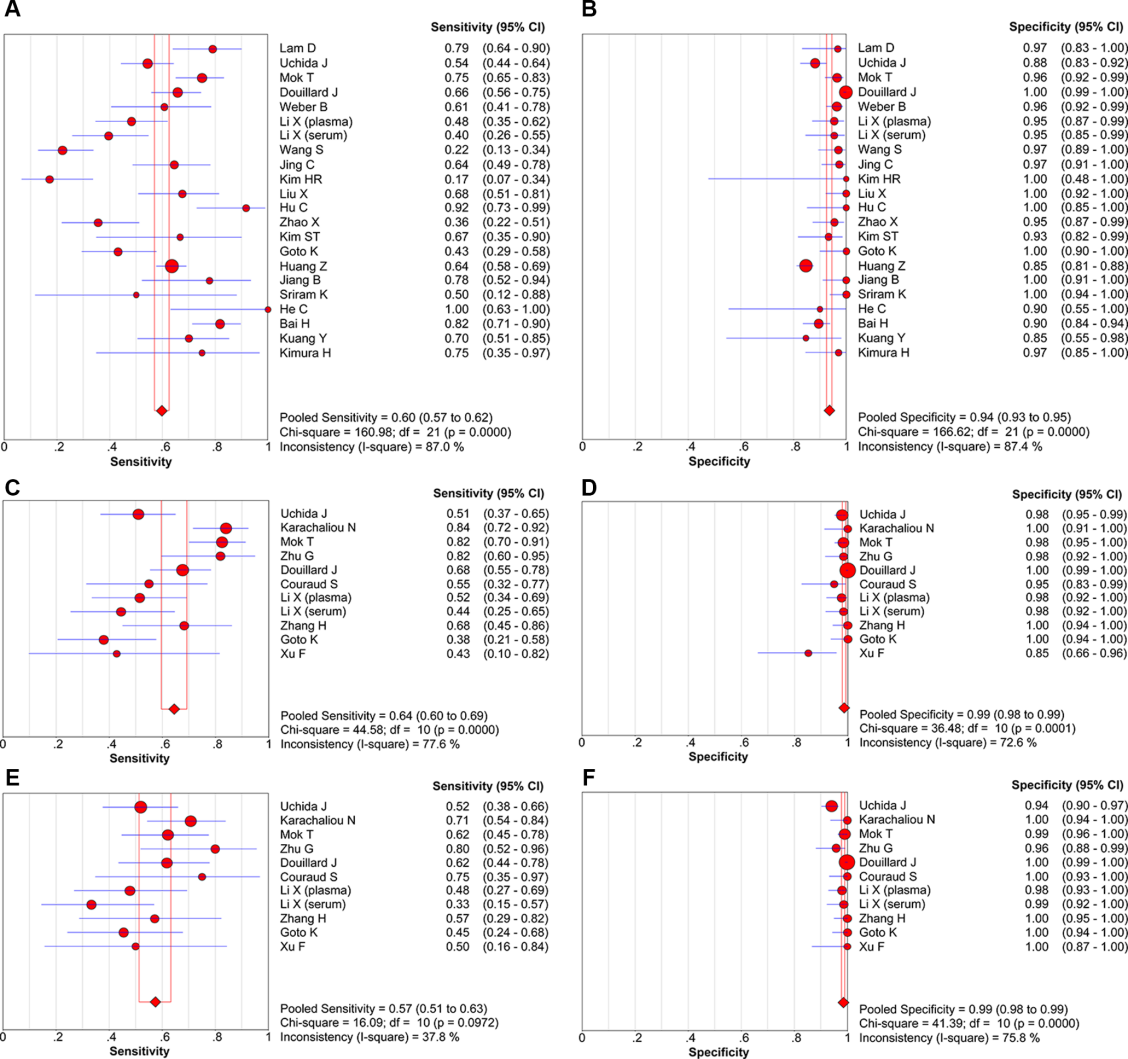
Forest plots of sensitivity and specificity of cfDNA for detection of all EGFR mutations (A, B), the exon 19 deletion (C, D), and the L858R point mutation (E, F)

**Figure 4:**
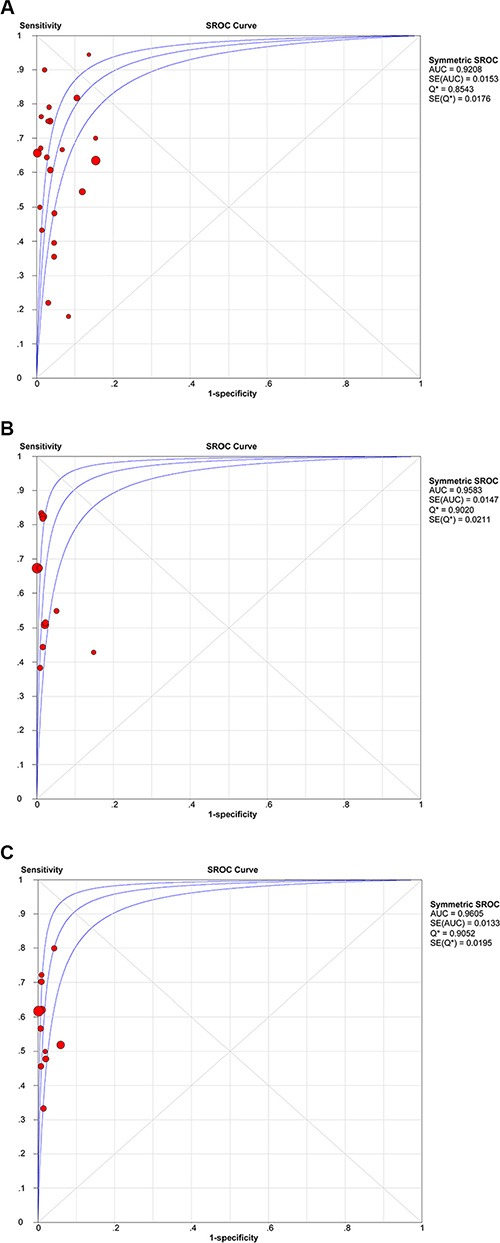
SROC curves of cfDNA for detection of all EGFR mutations (A), the exon 19 deletion (B), and the L858R point mutation (C)

Compared with NSCLC tumor tissues, the pooled sensitivity and specificity of cfDNA for the detection of EGFR mutation status were 0.60 (95% confidence intervals (95% CI) = 0.57–0.62) and 0.94 (95% CI = 0.93–0.95), respectively. The pooled sensitivity and specificity were 0.64 (95% CI = 0.60–0.69) and 0.99 (95% CI = 0.98–0.99) for detection of the exon 19 deletion, and 0.57 (95% CI = 0.51–0.63) and 0.99 (95% CI = 0.98–0.99) for detection of the L858R point mutation (Figure 3). positive likelihood ratio (PLR) and negative likelihood ratio (NLR) of cfDNA were 12.02 (95% CI = 7.71–18.74) and 0.41 (95% CI = 0.33–0.51) for detection of all mutations, 29.16 (95% CI = 12.82–66.29) and 0.39 (95% CI = 0.29–0.51) for detection of the exon 19 deletion, and 36.87 (95% CI = 16.17–84.09) and 0.44 (95% CI = 0.38–0.50) for detection of the L858R point mutation (Table 3). The summary receiver operating characteristic (SROC) curves showed that the areas under the curve (AUC) of cfDNA for detection of all EGFR mutations, the exon 19 deletion, and the L858R point mutation were 0.9208, 0.9583, and 0.9605, respectively (Figure 4).

**Table 3:** Subgroup analysis

	Study	Sensitivity	Specificity	PLR	NLR	DOR	AUC
**Mutation**							
All EGFR mutations	22	0.60 (0.57–0.62)	0.94 (0.93–0.95)	12.02 (7.71–18.74)	0.41 (0.33–0.51)	34.36 (19.75–59.76)	0.9208
Exon 19 deletion	11	0.64 (0.60–0.69)	0.99 (0.98–0.99)	29.16 (12.82–66.29)	0.39 (0.29–0.51)	84.74 (33.27 – 215.88)	0.9583
L858R point mutation	11	0.57 (0.51–0.63)	0.99 (0.98–0.99)	36.87 (16.17 – 84.09)	0.44 (0.38–0.50)	91.28 (37.51–222.10)	0.9605
**Blood type**							
Plasma	15	0.60 (0.57–0.63)	0.93 (0.92–0.94)	10.45 (6.37–17.14)	0.42 (0.32–0.54)	29.36 (15.60–55.26)	0.9146
Serum	7	0.56 (0.48–0.64)	0.98 (0.95–0.99)	20.37 (9.45–43.91)	0.40 (0.26–0.60)	45.42 (18.99–108.62)	0.9347
**Country**							
China	13	0.62(0.58–0.65)	0.91 (0.89–0.92)	11.19 (6.52–19.21)	0.37 (0.27–0.51)	34.55 (17.14–69.66)	0.9211
Japan	3	0.52 (0.44–0.60)	0.91 (0.87–0.94)	10.67 (2.40- 47.35)	0.51 (0.43–0.61)	24.23 (4.33–135.56)	0.8999
Korea	2	0.30 (0.17–0.45)	0.94 (0.83–0.99)	6.83 (2.40–19.45)	0.58 (0.13–2.63)	11.27 (1.03–123.54)	
Other	4	0.65 (0.57–0.72)	0.99 (0.98–0.99)	30.35 (4.84–190.29)	0.36 (0.30–0.45)	81.12 (12.05–546.05)	0.9569
**Sample size**							
≥ 90	11	0.59 (0.56–0.62)	0.93 (0.92–0.94)	10.73 (6.29–18.29)	0.45 (0.34–0.59)	26.41 (13.65–51.08)	0.9054
< 90	11	0.62 (0.56–0.68)	0.98 (0.95–0.99)	17.42 (9.64–31.50)	0.34 (0.22–0.54)	53.88 (24.63–117.84)	0.9422
**Detection method**							
PNA-LNA PCR clamp	2	0.76 (0.63–0.87)	0.95 (0.87–0.99)	16.95 (5.07–56.73)	0.26 (0.16–0.42)	59.25 (16.49–212.84)	
AS-PCR	2	0.72 (0.63–0.79)	0.96 (0.94–0.98)	20.02 (10.24–39.11)	0.32 (0.20–0.49)	65.99 (31.49–138.31)	
ARMS	8	0.51 (0.46–0.56)	0.99 (0.98–0.99)	17.80 (6.58–48.21)	0.48 (0.35–0.67)	40.39 (12.81–127.34)	0.9291
HRM	2	0.74 (0.62–0.84)	0.98 (0.93–1.00)	29.00 (8.14–103.26)	0.22 (0.06–0.79)	97.31 (25.78–367.40)	
ME-PCR	3	0.46 (0.33–0.59)	0.97 (0.93–0.99)	8.91 (3.81–20.85)	0.53 (0.27–1.05)	19.13 (6.50–56.33)	0.9111
DHPLC	2	0.67 (0.62–0.72)	0.86 (0.83–0.88)	5.48 (2.93–10.24)	0.31 (0.14–0.65)	18.34 (4.69–71.64)	

### Threshold effect and heterogeneity

Spearman correlation coefficients and *p* values were calculated to assess the threshold effect using Meta-DiSc meta-analysis software [40]. The Spearman correlation coefficients for all EGFR mutations, the exon 19 deletion, and the L858R point mutation were −0.018, −0.255, and −0.055, respectively, and the *p* values were 0.938, 0.450, and 0.873, respectively, indicating that the threshold effect was not significant. As shown in Figure 3, the heterogeneity caused by the non-threshold effect was high, so we conducted meta-regression analysis to detect the source of heterogeneity. However, the results showed that the country, study size, detection method, and blood type did not contribute to heterogeneity (Table 4).

**Table 4:** Meta-regression with the covariates

Covariates	All EGFR mutations					Exon 19 deletion					L858R point mutation				
	Coefficient	Standard error	P value	RDOR	95% CI	Coefficient	Standard error	P value	RDOR	95% CI	Coefficient	Standard error	P value	RDOR	95% CI
Country	0.005	0.2880	0.9852	1.01	0.55-1.85	0.151	0.6364	0.8217	1.16	0.23 – 5.97	0.376	0.4240	0.4158	1.46	0.49 - 4.33
Bloos type	0.182	0.8601	0.8354	1.2	0.19-7.43	−1.387	1.2545	0.3191	0.25	0.01 – 6.28	−1.297	0.9576	0.2335	0.27	0.02 – 3.20
Size	−0.753	0.7923	0.3560	0.47	0.09-2.53	−0.918	1.2378	0.4915	0.40	0.02 – 9.62	0.912	0.9504	0.3811	2.49	0.22 – 28.66
Method	0.011	0.1443	0.9414	1.01	0.74-1.37	0.268	0.4298	0.5592	1.31	0.43 – 3.95	0.232	0.3021	0.4774	1.26	0.58 – 2.74

EGFR: epidermal growth factor receptor; 95 % CI: 95 % confidence interval; RDOR: relative diagnostic odds ratios

## DISCUSSION

Although tumor tissue is the gold standard for detection of EGFR mutation status, major barriers exist in terms of acquisition and utility. To overcome the limitations of tissue biopsies, cfDNA can, in principle, provide the same genetic information as a tissue biopsy [11]. A number of studies have investigated the use of cfDNA for detection of the EGFR mutation status with varying results. Here, we performed a meta-analysis to evaluate the diagnostic accuracy of cfDNA for detection of EGFR mutations.

The pooled sensitivity and specificity of cfDNA for detection of EGFR mutations were 0.60 and 0.94, respectively. Several studies reported that differences in clinical outcomes were associated with different mutations. Lung cancer patients harboring the EGFR exon 19 deletion achieve longer survival following treatment with gefitinib or erlotinib, as compared to those with tumors harboring the L858R point mutation [8–10]. Additionally, the median overall survival (mOS) was shorter in patients with the L858R point mutation by cfDNA analysis than in those with the exon 19 deletion (13.7 vs. 30.0 months, respectively, *p* < 0.01) [39]. So, we also estimated the diagnostic accuracy of cfDNA for detection of the exon 19 deletion and the L858R point mutation. Our result showed that the pooled sensitivities of cfDNA for detection of the exon 19 deletion and the L858R point mutation were 0.64 and 0.57, and the pooled specificity were 0.99 and 0.99, respectively, indicating that cfDNA had a high degree of specificity, likely because mutations found in cfDNA are, in essence, integral agents of tumors that are defined by their presence in tumor DNA and absence in matched normal DNA [11].

cfDNA analysis is a noninvasive technique to predict patient response to EGFR-TKI treatment, dynamically assess treatment outcome, and facilitate early detection of resistance mutations. Que D et al. [41] reported that EGFR-TKI treatment significantly improved mOS in patients harboring the EGFR mutation in cfDNA than those that did not exhibit EGFR mutation (25.7 vs 13.5 months, respectively). Additionally, for EGFR mutations at baseline patients, Lee et al. [42] reported that the mOS was improved among patient with undetectable EGFR, as compared to detectable EGFR mutations in cfDNA (23.7 vs. 11.2 months) after TKI treatment for 2 months, in accordance with the findings of Mok et al. [15] who reported that the mOS for patients who continued to have detectable EGFR mutations at cycle 3 was 18.2 months and 31.9 months for patients without detectable mutations. The T790M point mutation is associated with acquired resistance to TKI therapy and reportedly occurs in about 50% of patients with disease progression after initial response to gefitinib or erlotinib [35]. As cfDNA had a high specificity to detect EGFR mutations, cfDNA might be a suitable noninvasive screening test to monitor T790M mutations during TKI treatment [35]. Lee et al. [42] found that 14 (28.6%) of 49 patients harbored the resistance mutation T790M in cfDNA during EGFR TKIs treatment, which is similar to the studies by Sakai et al. [43] and Sorensen et al. [44], in which the T790M mutation was detected in 21 (30%) of 75 and 9 (39%) of 23 blood samples from patients with clinical progressive disease. Most interestingly, several studies demonstrated that monitoring the EGFR mutations in cfDNA allows for the detection of the T790M mutation up to 344 days (range, 15–344 days) before radiographic documentation of disease progression [42, 44]. Additionally, Patients whose EGFR mutations switched from positive to negative after chemotherapy achieved a better partial response than patients with a reversal in mutation status [45], indicating that the high specificity of cfDNA could serve as an effective test to estimate the effect of chemotherapy.

The AUC is an established global measure of performance of diagnostic tests. According to the criteria, 0.9 < AUC < 1 indicates high diagnostic accuracy [46]. Our result showed that the AUCs of all EGFR mutations, the exon 19 deletion, and the L858R point mutation were 0.9208, 0.9583, and 0.9605, respectively, indicating high diagnostic accuracy of cfDNA. Likelihood ratios are alternative statistical parameters to summarize diagnostic accuracy and values > 10 and < 0.1 are considered to provide strong evidence to rule in or rule out diagnoses, respectively [47]. In the present study, the PLR was > 10, indicating that cfDNA accurately confirmed the presences of EGFR mutations (Table 3). Diagnostic odds ratio (DOR) is a single indicator of test performance that combines the strengths of sensitivity and specificity with the advantage of accuracy. The value of a DOR ranges from 0 to infinity, with higher values indicating better discriminatory test performance [48]. Our results showed that cfDNA had high diagnostic performance with DORs of 34.36, 84.74 and 91.28 for detection of all EGFR mutations, the exon 19 deletion, and the L858R point mutation. We used Spearman correlation coefficients and *p* values to assess the threshold effect, which is a major source of intra-study heterogeneity. The *p* values for all EGFR mutations, the exon 19 deletion, and the L858R point mutation were 0.938, 0.450, and 0.873, respectively, indicating that the threshold effect was not significant. Thus, meta-regression analysis was performed to detect the source of heterogeneity, but, unfortunately, none of the analyzed covariates was found to be the source of heterogeneity (Table 4).

Various methods can be used to detect EGFR mutations in cfDNA, such us allele-specific PCR (AS-PCR) [15], peptide nucleic acid-locked nucleic acid polymerase chain reaction (PNA-LNA-PCR) [18], amplification refractory mutation system (ARMS) [20], high resolution melting (HRM) [22], mutant-enriched (ME)-PCR [26], and denaturing high-performance liquid chromatography (DHPLC) [29]. Our results showed that the sensitivities of PNA-LNA PCR, AS-PCR, and HRM were higher than those of ARMS and ME-PCR, but the specificity of ARMS was the highest among the other tests. Analysis of methods for detection of EGFR mutations in plasma demonstrated that ARMS had highest specificity, as compared with the other methods [38]. Although cfDNA can be extracted from either plasma or serum, our results showed that cfDNA extracted from serum had higher diagnostic accuracy than that extracted from plasma (Table 3).

There were some limitations to this study that should be addressed. First, chemotherapy can change the EGFR status [45], which could lead to analytical inconsistencies between tissues and cfDNA in blood collected after treatment. Second, although we accessed the threshold effect and performed meta-regression analysis, high heterogeneity was detected, but none of the analyzed factors was found to be the source of the heterogeneity. Therefore, other factors, such as sex, smoking status, or tumor size may have been the cause of the observed heterogeneity. Third, publication bias was detected when the performance of cfDNA to detect the exon 19 deletion was analyzed; therefore, we conducted sensitivity analysis and found that the pooled results were not affected by the inclusion of individual studies.

In conclusion, cfDNA offers an effective and noninvasive method to detect EGFR mutation status in NSCLC. Due to its high specificity and noninvasive characteristics, cfDNA analysis presents a promising method to screen for mutations in NSCLC and predict patient response to EGFR-TKI treatment, dynamically assess treatment outcome, and facilitate early detection of resistance mutations.

## MATERIALS AND METHODS

### Search strategy

A comprehensive search of the PubMed (http://www.ncbi.nlm.nih.gov/pubmed) and Google Scholar (http://www.scholar.google.com/) databases using the keywords “cell free DNA OR circulating DNA OR circulating tumor DNA OR serum DNA OR plasma DNA” AND “lung cancer OR non-small cell lung cancer” AND “EGFR OR Epidermal Growth Factor Receptor OR erbB1” was conducted to identify relevant studies published before September 28, 2015. In addition, the references from the retrieved articles that matched our inclusion criteria were manually searched.

### Inclusion and exclusion criteria

The inclusion criteria for studies were as follows: (a) a histopathological diagnosis of NSCLC; (b) matched tissue and cfDNA sample; (c) identification of EGFR mutation status both in tissue and cfDNA; (d) sufficient data to construct a diagnostic 2 × 2 table; and (e) enrollment of at least 15 patients. Studies were excluded if they were: (a) not written in English; (b) tumor tissue and blood samples were not paired; or (c) case reports or reviews. Two of the authors (X.Q. and J.L.) read the titles and abstracts independently, and excluded studies that did not meet the inclusion criteria. Then, the full texts were screened to determine if they met the inclusion criteria.

### Data extraction

Two independent reviewers (YH.S. and MF.W.) assessed the articles. The name of the first author, year of publication, country, histologic type, tumor stage, distribution of age and sex, techniques used for EGFR mutation detection in cfDNA, serum or plasma, and true positive (TP), false positive (FP), false negative (FN), and true negative (TN) rates were collected from eligible studies. When EGFR mutations were detected by multiple methods, the method with the best sensitivity or specificity was extracted.

### Quality assessment

Quality assessment of diagnostic accuracy studies 2 (QUADAS-2) is a revised tool for the quality assessment of diagnostic accuracy studies [49]. The QUADAS-2 comprises four domains: patient selection, index test, reference standard, and flow and timing. Signaling questions are included to help judge risk of bias as “low,” “high,” or “unclear.”

### Statistical analysis

The threshold effect was estimated with Meta-DiSc meta-analysis software [40]. A probability (*p*) value < 0.05 was considered to reflect a significant threshold effect. Heterogeneity due to a non-threshold effect was determined using the *Q* test and the inconsistency index (*I*^2^) test with *p* ≤ 0.05 and *I*^2^ ≥ 50% indicating significant heterogeneity. Meta-regression analysis was conducted to detect the source of heterogeneity. According to the heterogeneity test results, a random or fixed model was used to pool the sensitivity/specificity rates, PLR, NLR, DOR, and corresponding 95% CI. SROC and AUC were also calculated. Publication bias and sensitivity analyses were performed using STATA software (version 11.0; StataCorp LP, College Station, TX, USA), while all other analyses were performed using Meta-DiSc (version 1.4) meta-analysis software [40].
